# Development of Step-Count Cut Points for School-Day Vigorous Physical Activity

**DOI:** 10.1155/2018/9717848

**Published:** 2018-03-28

**Authors:** Ryan D. Burns, Timothy A. Brusseau, You Fu, Peng Zhang

**Affiliations:** ^1^Department of Health, Kinesiology, and Recreation, University of Utah, Salt Lake City, UT, USA; ^2^School of Community Health Sciences, University of Nevada, Reno, Reno, NV, USA; ^3^Department of Exercise Science, East Stroudsburg University, East Stroudsburg, PA, USA

## Abstract

**Background:**

No study has established step-count cut points for varying amounts of accelerometer-assessed vigorous physical activity (VPA) accrued during the school day in children. The purpose of this study was to establish step-count cut points for discriminating children meeting VPA in 5 minutes, 10 minutes, 15 minutes, and 20 minutes per 7-hour school day.

**Methods:**

Participants were a convenience sample of 1,053 children (mean age = 8.4 (1.8) years) recruited from 5 schools from the Mountain West region of the USA. Data within students were observed across multiple semesters totaling 2,119 separate observations. Step counts and time in VPA were assessed using ActiGraph wGT3X-BT triaxial accelerometers that were worn during the entirety of a 7-hour school day for one school week. Average censored step counts and minutes in VPA were calculated across 3 to 5 days. Receiver operating characteristic (ROC) curves were employed to derive step counts via calculation of the maximum Youden *J* statistic.

**Results:**

Area-under-the-curve (AUC) scores ranged from AUC = 0.81 (95% CI: 0.78–0.83; *p* < 0.001) for meeting at least 5 minutes of VPA to AUC = 0.94 (95% CI: 0.88–1.00, *p* < 0.001) for meeting at least 20 minutes of VPA. Approximately 3,460 steps best discriminated children meeting at least 5 minutes of VPA (sensitivity = 74.0%, specificity = 74.0%, and accuracy = 74.1%) and approximately 5,628 steps best discriminated children meeting at least 20 minutes per day of VPA (sensitivity = 85.7%, specificity = 95.1%, and accuracy = 95.1%).

**Conclusion:**

Step counts can discriminate with reasonable accuracy children that meet at least 5 minutes of school-day VPA and with strong accuracy children that meet 20 minutes of school-day VPA.

## 1. Introduction

Higher levels of vigorous physical activity (VPA) have been more strongly linked to lower mortality in adults and improved health-related fitness in youth compared to moderate physical activity [1, 2]. By convention, physical activity recommendations and research in physical activity surveillance and intervention efficacy use moderate-to-vigorous physical activity (MVPA) as the primary metric for assessment [3]; however research supports there being stronger independent correlations between VPA and health in the pediatric population [4, 5]. Unfortunately, age related declines in VPA appear to be greater compared to declines in moderate physical activity [6]. Although the “moderate” physical activity component of MVPA may relate to improved health outcomes in high-risk youth (e.g., those with obesity), adults, and the geriatric population [7, 8], in lower-risk children, moderate physical activity has shown in many studies to be only a modest predictor of health-related fitness and cardiometabolic health outcomes [4]. This is not to deny moderate physical activity's role in emotional wellbeing, cognitive functioning, and other benefits [9, 10], but this is to communicate that it is through increasing VPA that there is a sufficient physiological response needed to improve cardiorespiratory endurance and to yield large energy expenditures needed to maintain or improve body composition [11, 12].

Because children spend a majority of their waking hours in school, physical activity surveillance and physical activity promotion in primary and secondary school settings have been an increasingly popular line of research [13, 14]. VPA may be especially important during school hours because of the long bouts of sedentary behavior that often accompany academic classes and limited time windows during the school day for PA engagement. Unfortunately, because of the expense of accelerometers and the time and resources needed to process and download accelerometer count data, physical activity assessment using large samples of children may not be feasible for many researchers and practitioners. Although the use of accelerometers is preferable in most contexts because of its ability to characterize intensity using validated count cut points, pedometer step counts offer an alternative method to assess physical activity [15]. Pedometers are inexpensive devices that record step counts and can provide meaningful information regarding children's ambulatory physical activity [16]. Pedometers can also be administered to large samples of children concurrently, with significantly less cost, time, and resources needed compared to accelerometers. Despite this, the major limitation of using step counts (pedometers) for physical activity assessment is the inability to characterize physical activity intensity [17]. Thus, determining step counts relating to various levels of physical activity will be useful for researchers, physical educators, and health practitioners when assessing large samples of children within school settings. There has been published research establishing step counts associated with MVPA [18–20]; however no study to date has established step-count cut points discriminating children meeting various levels of VPA during school hours. Therefore, the purpose of this study was to develop step-count cut points discriminating 5 minutes of VPA, 10 minutes of VPA, 15 minutes of VPA, and 20 minutes of VPA during a 7-hour school day in children from the first through 6th grades.

## 2. Methods

### 2.1. Participants

Participants were a convenience sample of 1,053 children (mean age = 8.4 (1.8) years; 525 girls, 528 boys) recruited from 5 schools from the Mountain West region of the USA. Participants were recruited from the first through 6th grades. Physical activity data within students were observed across multiple semesters totaling 2,247 separate observations. Because of missing or invalid data, the final sample consisted of 2,119 separate observations (94.3% of original sample; 8.5 (1.9) years; 1,041 girls, 1,078 boys). The final sample distribution per grade level included 533 first-grade observations, 521 second-grade observations, 443 third-grade observations, 343 fourth-grade observations, 172 fifth-grade observations, and 107 sixth-grade observations. Data were collected across three school years between 2014 and 2017. All participants were enrolled in schools receiving a Comprehensive School Physical Activity Program that was funded by the US Department of Education. The 5 schools from which all participants were recruited were considered of low income and “Title 1” and thus were receiving supplemental government financial assistance.

### 2.2. Procedures

School-day steps and time in VPA were assessed using ActiGraph wGT3X-BT triaxial accelerometers (Pensacola, FL, USA). The step-count function in ActiGraph models has shown evidence for moderately high degree of criterion validity with accelerometer counts (*r* = 0.82) and convergent validity with Omron pedometer step counts (*r* = 0.89) in children [21]. Low force steps were censored because ActiGraph accelerometer step counts are more sensitive to low force acceleration compared to research-grade pedometers [19]. Therefore, accelerometer censored step counts may more strongly agree with pedometer steps. Step counts were disregarded if the associated accelerometer counts were less than 500 counts/minute. The low-frequency extension was not used. Each participant in the original sample (*N* = 2,247) was instructed to wear the accelerometer for 5 school days (Monday through Friday) between the hours of 8 am and 3 pm with no included non-wear time. Accelerometers were worn on the right hip at the level of the iliac crest, aligned with the kneecaps. Classroom teachers, physical educators, and members of the research team ensured that the devices were worn during the entirety of the school day. A valid day for accelerometers was determined to be at least 7 valid hours out of total wear time for at least 3 days of the school week. Approximately 2,119 observations met these criteria (94.3% of the original sample) and therefore were subsequently included in the data analysis.

Accelerometer data were recorded in 15-second epochs at 100 Hertz and subsequently processed using the Evenson et al. [22] cut points. Evenson et al. [22] cut points are often used to classify physical activity intensity in children and adolescents because of established strong criterion-referenced energy expenditure agreement with indirect calorimetry [23]. The epochs during the school day were classified as sedentary, light, moderate, or vigorous physical activity. The ActiLife 6.11.5 software program (Pensacola, FL, USA) was used to initialize, download, process, and store accelerometer data.

Data were collected across 3 academic school years between 2014 and 2017 (5 semesters total). Data were collected both in the fall and spring semesters across the 5 schools. Schools involved in this study were part of the same school district and therefore had similar bell schedules and all were characterized by a 7-hour school day. Accelerometer step count and count data used in the subsequent analysis included the school-day averages across one school week (3–5 days).

### 2.3. Statistical Analysis

Differences between the sexes on school-day step counts, MVPA, and VPA were examined using independent *t*-tests. The average time in school-day VPA was stratified into a binary classification scheme for 5 minutes of school-day VPA, 10 minutes of school-day VPA, 15 minutes of school-day VPA, and 20 minutes of school-day VPA. Receiver operating characteristic (ROC) curves were developed to determine the number of school-day step counts needed to discriminate students who did and who did not achieve various VPA levels during school hours. Separate ROC curves were developed for each VPA cut point. To keep sample sizes large, ROC curves were not developed for more specific age or age-sex groups. Overall diagnostic power was determined using the area under the curve (AUC). The sex differences between AUC scores were examined using STATA's “roccomp” command. AUC scores of ≥0.90 were considered excellent, 0.80–0.89 good, 0.70–0.79 fair, and <0.70 poor [24]. Step-count cut points for each ROC curve were determined using the maximum Youden *J* statistic, which was calculated using STATA's “senspec” command and is the point on the ROC curve that maximizes the sum of sensitivity and specificity (*J* max = max⁡((sensitivity + specificity) − 1)). Sensitivity was the probability that a student achieved a VPA cut point based on step counts (T^+^) given that he or she actually did achieve the VPA cut point via accelerometer counts (*D*^+^) or *P*(*T*^+^∣*D*^+^). Sensitivity is synonymous with the probability of achieving a true positive. Specificity was the probability that a student did not achieve a VPA cut point (*T*^−^) based on step counts, given that he or she did not achieve the VPA cut point via accelerometer counts, *P*(*T*^−^∣*D*^−^), or a true negative [25]. Maximizing sensitivity and specificity scores associates with the datum closest to (0, 1) on the ROC curve and is a step-count cut point that is likely to yield high classification accuracy compared to other cut points. For each derived step-count cut point, classification accuracy (i.e., the percentage of children correctly classified having met or not met a respective VPA cut point) was calculated. The aforementioned methodology was similar to that used in Burns et al. (2016) [18]. Alpha level was set at *p* ≤ 0.05 and all analyses were carried out using STATA v14.0 statistical software package (College Station, TX, USA).

## 3. Results

The descriptive statistics for the total sample and within sex groups are displayed in Table 1. Boys displayed a higher number of school-day steps (mean difference = 556 steps, *p* < 0.001, Cohen's *d* = 0.39). However, there were no differences between the sexes in minutes of school-day MVPA or VPA. There were 726 weekly observations (34.2% of total sample) that met at least 5 minutes per school-day VPA, 157 weekly observations (7.4% of total sample) meeting at least 10 minutes of school-day VPA, 33 weekly observations (1.6% of total sample) meeting at least 15 minutes of school-day VPA, and 10 weekly observations (0.5% of total sample) meeting 20 minutes of school-day VPA.

**Figure 1:**
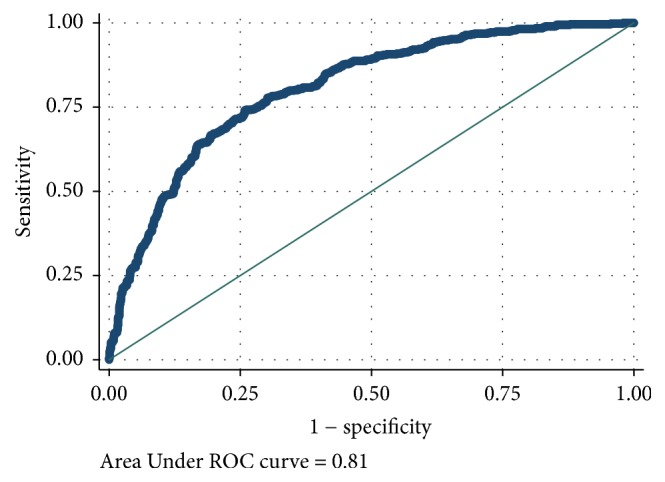
Receiver operating characteristic curve for step counts discriminating 5 minutes of school-day vigorous physical activity.

**Figure 2:**
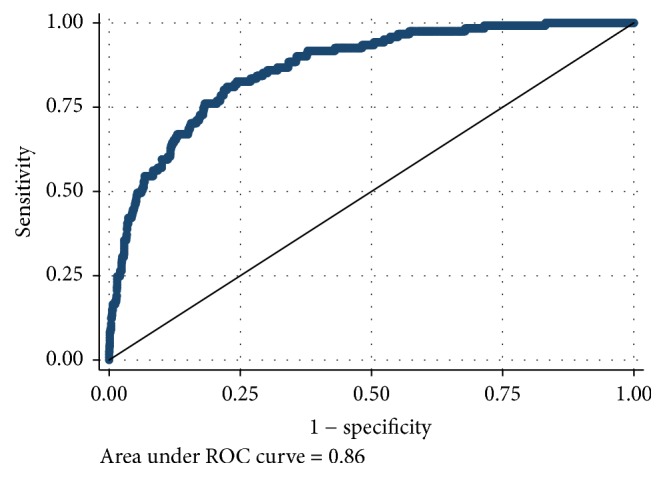
Receiver operating characteristic curve sex discordance for step counts discriminating 10 minutes of school-day vigorous physical activity.

**Figure 3:**
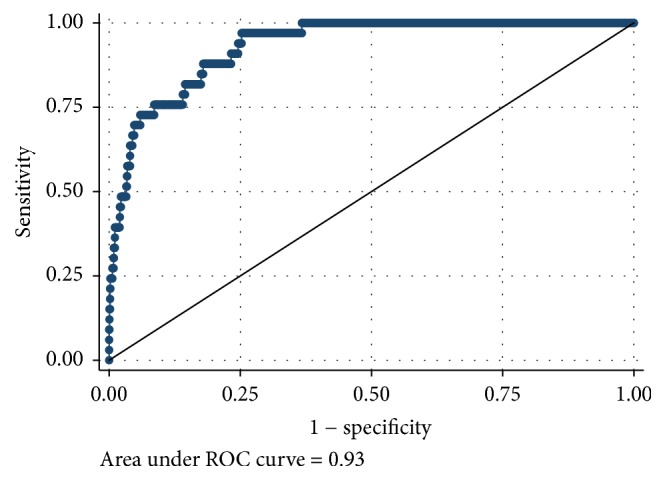
Receiver operating characteristic curve for step counts discriminating 15 minutes of school-day vigorous physical activity.

**Figure 4:**
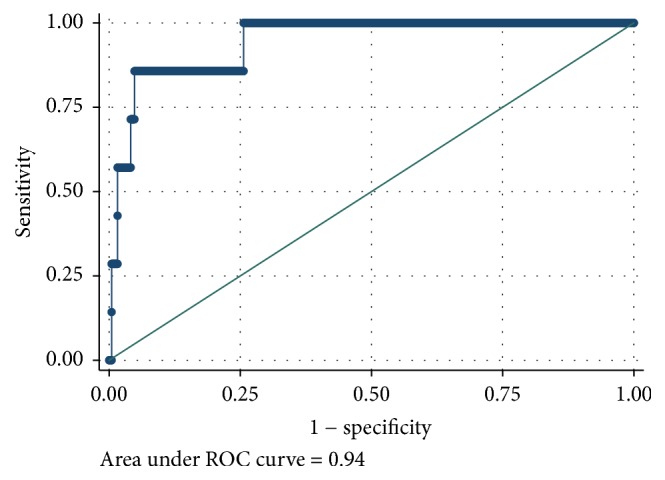
Receiver operating characteristic curve sex discordance for step counts discriminating 20 minutes of school-day vigorous physical activity.

Table 2 reports the results of the ROC analyses for each VPA cut point ranging from 5 minutes of school-day VPA to 20 minutes of school-day VPA. AUC scores ranged from good to excellent. The AUCs were AUC = 0.81 (95% CI: 0.78–0.83; *p* < 0.001) for meeting at least 5 minutes of VPA, AUC = 0.86 (95% CI: 0.83–0.89; *p* < 0.001) for meeting at least 10 minutes of VPA, AUC = 0.93 (95% CI: 0.89–0.96; *p* < 0.001) for meeting at least 15 minutes of VPA, and AUC = 0.94 (95% CI: 0.88–1.00, *p* < 0.001) for meeting at least 20 minutes of VPA. There were no statistical differences between sexes on the AUCs for any VPA cut point. Figures 1–4 display the ROC curves for each VPA cut point. Step counts discriminated with reasonable accuracy students who achieved 5 minutes of VPA (3,460 steps; sensitivity = 74.0%; specificity = 74.0%; 74.1% accuracy), 10 minutes of VPA (4,133 steps; sensitivity = 81.0%; specificity = 77.5%; 77.8% accuracy), and 15 minutes of VPA (4,133 steps; sensitivity = 97.0%; specificity = 74.8%; 75.2% accuracy) and with strong accuracy those students who achieved 20 minutes of VPA (5,628 steps; sensitivity = 85.7%; specificity = 95.1%; 95.1% accuracy).

## 4. Discussion

The purpose of this study was to develop step-count cut points that discriminate children meeting various levels of school-day VPA. The results showed that step counts were able to discriminate with reasonable accuracy children meeting 5 minutes of school-day VPA through 15 minutes of school-day VPA and with strong accuracy children meeting 20 minutes of school-day VPA. These cut points were derived across a 7-hour school day. Because past research has shown the importance of increasing VPA in the pediatric population, the derived cut points can be used for physical activity surveillance in school settings for clinical and research purposes in the absence of accelerometers, because of either unavailability or logistical limitations. Implications for health-related fitness, how the derived VPA cut points compare to prior established MVPA step-count cut points, and possible applications in school settings are discussed further.

Pedometers have strong practical utility in the pediatric population because they are reliable and inexpensive and the output is simple to understand [26]. Understanding step-count cut points for varying amounts of accelerometer-assessed VPA fills in the knowledge gap of pedometer's inability to characterize physical activity intensity. The results of this study can facilitate researchers and health professionals to evaluate the physical activity intensity levels during the school day and can determine activities that accumulate VPA levels, which can elicit greater benefits in cardiorespiratory fitness and body composition [26]. In pediatric population, VPA has been shown to significantly improve cardiorespiratory endurance [27]. VPA was also effective at lowering the resting blood pressure in overweight and obese adolescents [28]. Interventions aimed at increasing VPA yielded significant improvements in VO_2  Peak_ and excess postexercise oxygen consumption [29]. Additionally, submaximal heart rate, resting pulmonary function, and ventilatory response to exercise were also improved following bouts of VPA [30, 31]. Tjønna et al. [32] reported greater improvements in blood glucose in a high intensity exercise group compared to a control group in a sample of overweight adolescents. These established beneficial effects of VPA indicate that VPA is an effective option to improve the health-related physical fitness of children. Additionally, increasing VPA may be a time-efficient strategy that can meet the children's inherent preference for physical activity [28]. The step-count cut points for VPA established from the study strengthen pedometer (step counts) utility on promoting children's health. Studies using objectively assessed physical activities have demonstrated that VPA was more closely associated with body composition compared to physical activity of lower intensities [33, 34]. Wittmeier and colleagues [35] revealed that children (aged 8–11 years) who performed less than 5 min/day of VPA were 4.0 times more likely to have greater than 20% body fat and 5.2 times more likely to be classified as overweight compared to children performing greater than 15 min/day of VPA. Carson et al. [28] examined longitudinal associations between different physical activity intensities and cardiometabolic risk factors in a sample of Canadian youth (aged 9–15 years) over a period of two years. The results showed that VPA was negatively associated with body composition while the participants significantly reduced their BMI *z*-scores and their waist circumference level in a dose-response manner. Finally, intervention studies have demonstrated that obese adolescents who spent the most weekly time engaged in VPA tended to be those who decreased the most in body fat [36]. Indeed, physical activity or exercise of a vigorous intensity (≥6 METs) incurs greater energy expenditures compared to moderate intensity (3–5.9 METs) performed for the same duration [37]. Despite the aforementioned evidence, physical activity volume is often not controlled for when comparing VPA to physical activity of lower intensities; therefore, it is unknown whether the greater benefits of VPA would hold if physical activity volume (e.g., MET-minutes per week, estimated energy expenditure) were constant. Future research needs to control for the potential confounding of physical activity volume when exploring health effect discordance among levels of physical activity intensity in children.

The practical utility of the derived cut points from this study may be strong in school settings. Tudor-Locke et al. [38] found that children average approximately 13,000 (boys) and 12,000 (girls) of accelerometer step counts per day, with censored steps reducing these amounts by approximately 2,600 steps. Adams et al. [19] showed that children needed to accumulate approximately 9,000 pedometer-scaled steps in order to meet the 60-minute-per-day MVPA guideline. The children in the current sample averaged approximately one-third (3,319 steps) of these recommended total day steps when it is recommended that children accrue at least one-half of total day physical activity during school hours [39]. Despite this low sample average for school-day step counts, the data yielded strong evidence to discriminate children meeting various levels of VPA. Given past research showing the discordance in health outcomes in children achieving 5 minutes per day of VPA and 15 minutes per day of VPA (35), accruing 4,000–5,000 steps (one-half of the daily recommended guideline) discriminates children meeting higher VPA cut points and aligns with current recommendation from the Institutes of Medicine recommending that children should accumulate one-half of physical activity guidelines during school. These derived cut points are lower than those found in other work establishing pedometer cut points for school-day MVPA (approximately 5,505 steps). This phenomenon may have been due to the inclusion of both moderate physical activity and VPA in the derived cut points in previous studies [18]. Both cut points (derived for MVPA and VPA) should be considered valid depending on the physical activity intensity that a researcher or practitioner aims to assess during the school day.

Accumulating one-half of pediatric physical activity guidelines during school hours seems to align well with quantitative empirical research establishing pedometer step counts for total day and school-day physical activity. Because the derived step-count cut points for higher amounts of VPA is greater than 4,000 and 5,000 steps for 15 minutes of VPA and 20 minutes of VPA, respectively, the message of accruing this number of steps counts should be a public health message for schools. Given the empirical research suggesting that 9,000 censored steps are an accurate cut point for total day MVPA in children [19], establishing one-half of this amount during school should be an easily understood goal for youth. The current study developed the step-count cut points using VPA as the criterion because of the sporadic high intensity movements young children often participate in during active play [40].

Despite the findings of the current study, analyses were not conducted within specific age and sex groups. Because of this, the results are potentially confounded by body size, specifically height, contributing to discordances in stride length across age and sex groups. Confounding may be present because stride length is inversely related to steps taken within a given time frame. Taller students may take fewer steps over a respective physical activity duration compared to shorter children. Future research developing cut points in developing youth should take into account a child's or adolescent's stride length to derive potentially more valid cut points.

There are other limitations to this study that must be considered before the results can be generalized. The sample consisted of first-grade through 6th-grade children recruited from schools located in the Mountain West region of the USA; therefore, the results are questionable if generalized to younger or older age groups or to populations within different geographical regions. Second, step counts were recorded using the ActiGraph accelerometer; the results may have differed if a separate pedometer monitor was used to record step counts concurrently with the accelerometer counts. Third, as stated previously, there may have been different results if the analyses were conducted on separate age groups to partially account for physical development, especially height as this may affect participant stride length. Fourth, data were collected across a 7-hour school day; therefore step-count cut points may differ if a longer or shorter bell schedule was used. Fifth, physical activity volume was not controlled for (e.g., MET-minutes per week, estimated energy expenditure) and moderate physical activity and light physical activity were not assessed, which undoubtedly contributes to step-count totals during the school day. Additionally, few students met the 15-minute-per-day and 20-minute-per-day VPA cut points; the validity of the results would be stronger if a greater proportion of the total sample were in these strata. Finally, data were collected across different semesters; therefore weather may have confounded the results due to its potential influence on outside play during recess or physical education.

## 5. Conclusions

In conclusion, step counts discriminate with reasonable accuracy children meeting VPA ranging from 5 to 15 minutes per 7-hour school day and with strong accuracy children meeting 20 minutes of school-day VPA per 7-hour school day. This is the first study to associate step counts with varying amounts of VPA accrued during the school day and may have several applications for clinical practice, surveillance, and intervention research. Because of the strong health benefits of VPA in the pediatric population, the use of step counts to discriminate children meeting various levels of the construct may provide further specificity and efficiency for school-based physical activity assessment.

## Figures and Tables

**Table 1:** Descriptive statistics for separate weekly data observations (means (standard deviations)).

	Total sample (*N* = 2,119)	Girls (*n* = 1,041)	Boys (*n* = 1,078)
Age (years)	8.5 (1.9)	8.3 (1.8)	8.6 (2.0)
School-day steps	3,319 (1328)	3,211 (1243)	**3,767 (1433)**
School-day MVPA (minutes)	31.7 (14.3)	29.9 (12.9)	33.1 (15.9)
School-day VPA (minutes)	4.7 (4.4)	4.2 (4.3)	4.9 (4.5)

*Note.* MVPA stands for moderate-to-vigorous physical activity; VPA stands for vigorous physical activity; bold indicates statistically significant differences between sexes, *p* < 0.05.

**Table 2:** Receiver operating characteristic curve step-count cut points across various levels of school-day vigorous physical activity.

VPA cut point	Step-count cut point	Sensitivity	Specificity	Maximum Youden's *J* Statistic	Accuracy
5 minutes of VPA	3,460	74.0%	74.0%	0.48	74.1%
10 minutes of VPA	4,133	81.0%	77.5%	0.56	77.8%
15 minutes of VPA	4,133	97.0%	74.8%	0.71	75.2%
20 minutes of VPA	5,628	85.7%	95.1%	0.81	95.1%

*Note.* VPA stands for vigorous physical activity.

## Abbreviations

AUC: Area under the curve
CSPAP: Comprehensive School Physical Activity Program
MVPA: Moderate-to-vigorous physical activity
VPA: Vigorous physical activity
ROC: Receiver operating characteristic curve.
